# Coherently driven quantum features using a linear optics-based polarization-basis control

**DOI:** 10.1038/s41598-023-40181-x

**Published:** 2023-08-09

**Authors:** Byoung S. Ham

**Affiliations:** School of Electrical Engineering and Computer Science, Gwangju Institute of Science and Technology, 123 Chumdangwagi-ro, Buk-gu, Gwangju, 61005 South Korea

**Keywords:** Quantum mechanics, Single photons and quantum effects

## Abstract

Quantum entanglement generation is generally known to be impossible by any classical means. According to Poisson statistics, coherent photons are not considered quantum particles due to the bunching phenomenon. Recently, a coherence approach has been applied for quantum correlations such as the Hong–Ou–Mandel (HOM) effect, Franson-type nonlocal correlation, and delayed-choice quantum eraser to understand the mysterious quantum features. In the coherence approach, the quantum correlation has been now understood as a direct result of selective measurements between product bases of phase-coherent photons. Especially in the HOM interpretation, it has been understood that a fixed sum-phase relation between paired photons is the bedrock of quantum entanglement. Here, a coherently excited HOM model is proposed, analyzed, and discussed for the fundamental physics of two-photon correlation using linear optics-based polarization-basis control. For this, polarization-frequency correlation in a Mach–Zehnder interferometer is coherently excited using synchronized acousto-optic modulators, where polarization-basis control is conducted via a selective measurement process of the heterodyne signals. Like quantum operator-based destructive interference in the HOM theory, a perfectly coherent analysis shows the same HOM effects of the paired coherent photons on a beam splitter, whereas individual output intensities are uniform.

## Introduction

Over the last several decades, quantum entanglement has been intensively studied for the weird quantum phenomena that cannot be obtained by classical physics^1–9^. The ‘weird’ quantum features are due to our limited understanding of quantum entanglement, as Einstein raised a fundamental question on nonlocal realism^1^. An intuitive answer to the impossible quantum feature by classical physics can be found in the uncontrolled tensor products of two bipartite particles, resulting in the classical lower bound in intensity correlation^10^. As shown for the self-interference of a single photon^11^, the wave-particle duality has been a main issue in quantum mechanics to understand the mysterious quantum superposition^12,13^. Here, a contradictory quantum feature driven by coherence optics is presented for the ‘weird’ quantum features using a polarization-basis control of coherent photons. As a result, the quantum feature of photon bunching of the Hong–Ou–Mandel (HOM) effects^14^ is analytically demonstrated for the coincidence detection of coherent photons from a beam splitter (BS), whereas output ports show a uniform intensity. The path-length dependent coherence effect is completely removed for the coherently derived HOM effects. Heterodyne-based^15^ and independent light-based HOM effects have also been observed, even though their physical understanding is limited^15,16^.

Recently, a coherence approach^17–20^ has been applied for the quantum features based on entangled photon pairs generated from the spontaneous emission parametric down-conversion (SPDC) process^21,22^ to understand their basic physics of the HOM effect^14^, Franson-type nonlocal correlation^23–25^, and delayed-choice quantum eraser^26–29^. On the contrary to conventional particle nature-based understanding, the nonlocal quantum feature between space-like separated photons originates in phase coherence-based basis-product modification resulting from coincidence detection^18,19^. This phase coherence commonly applies to both distinguishable (particle nature) and indistinguishable (wave nature) characteristics of a single photon, where a specific phase relationship between the paired photons has already been derived from both HOM^17^ and delayed-choice quantum eraser^19^. Such a phase relation is provided by a fixed sum phase between paired photons according to the phase-matching condition of second-order nonlinear optics^28,30^. These are the backgrounds of the present coherence approach to the coherence quantum feature using polarization-basis modification of coherent photons to understand otherwise the ‘weird’ quantum phenomenon.

**Figure 1:**
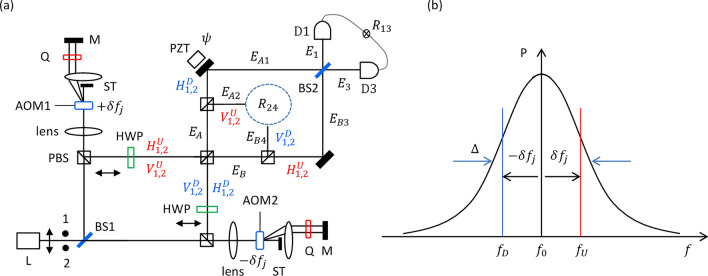
Schematic of coherence entangled photon-pair generation from an attenuated laser. (**a**) Schematic of polarization-basis separation. (**b**) An AOM-generated frequency-path correlated photon pair in (**a**). *BS* non-polarizing beam splitter, *AOM* acousto-optic modulator, *D* single photon detector, *HWP* half-wave plate, *M* mirror, *PBS* polarizing beam splitter, *PZT* piezo-electric transducer, *Q* quarter-wave plate, *ST* beam stopper, ${R}_{13}$ heterodyne two-photon coincidence detection. Dots 1 and 2 indicate identical single photons at vertical polarization.

Compared with conventional nonlinear optics-based methods suffering from entanglement degradation by imperfectness in the generation and collection of photon pairs needed for error correction, distillation, and/or purification for potential applications^31–33^, the present method is much more stable and robust due to coherence optics with a matured MZI stabilization technique, where the major error source is from air turbulence, temperature, and mechanical vibrations^34^. The coherence time (length) between polarization-frequency correlated photons in Fig. 1 is determined by the linewidth of the laser L, which can be extremely narrower compared with those based on nonlinear optics such as SPDC. However, the fundamental error of the coherent photons is due to Poisson statistics, resulting in an inevitable ~ 1% statistical error^35^. Regarding coherence manipulations of the HOM effects in an MZI, most conventional literature has been focused not on the coherent photons but on the coherence manipulation between entangled photons, resulting in the first-order intensity correlation-like features^36,37^. Although some coherent photon-based HOM effects have been experimentally demonstrated^15,16^, their physical understandings have been vague until recently^17^. On contrary to conventional works^14–16,36,37^, the present paper is the first proposal of coherent photon-based quantum correlation using linear optics. For this, polarization-basis control is the key to understanding the selective measurement-based quantum features.

## Results

Figure 1a shows the schematic of the coherently derived quantum features using an attenuated laser via polarization-basis control. To provide random polarization bases of a single photon, the laser L is followed by a $22.5^\circ$-rotated half-wave plate (HWP). Using neutral density filters, the randomly polarized photons are maintained at a low mean photon number state, satisfying independent measurement-based statistics^35^. For the phase-matched coherent photon pairs, a set of acousto-optic modulators (AOMs) are used in both paths of the noninterfering Mach–Zehnder interferometer (NMZI), where the AOMs are synchronized and oppositely scanned each other for a given bandwidth $\Delta$. For the polarization-basis separation of NMZI output photon pairs, an additional polarizing beam splitter (PBS) is added to each output port of the NMZI. Due to the coincidence detection of a photon pair, two (independent) polarization-correlated photon pairs, e.g., horizontal (H)–H and vertical (V)–V photon pairs in Table 1 (color matched) are provided independently. For the proof of principle, the polarization-correlated photon pairs are tested on a BS for the quantum feature of the HOM effects.

**Table 1:** A total of 16 possible ways to distribute photon pairs in Fig. 1a.

	Photon 1-up; photon 2-down				Photon 1-down; photon 2-up			
$\mathrm{Up}$	${H}_{1}^{U}$	${H}_{1}^{U}$	${V}_{1}^{U}$	${V}_{1}^{U}$	${H}_{2}^{U}$	${H}_{2}^{U}$	${V}_{2}^{U}$	${V}_{2}^{U}$
$\mathrm{Down}$	${H}_{2}^{D}$	${V}_{2}^{D}$	${H}_{2}^{D}$	${V}_{2}^{D}$	${H}_{1}^{D}$	${V}_{1}^{D}$	${H}_{1}^{D}$	${V}_{1}^{D}$

	Photon 1-up; Photon 2-up				Photon 1-down; Photon 2-down			
$\mathrm{Up}$	${{H}_{1}^{U}-H}_{2}^{U}$	${{H}_{1}^{U}-V}_{2}^{U}$	${{V}_{1}^{U}-V}_{2}^{U}$	${{V}_{1}^{U}-H}_{2}^{U}$	$0$	$0$	$0$	$0$
$\mathrm{Down}$	$0$	$0$	$0$	$0$	${{H}_{1}^{D}-H}_{2}^{D}$	${{H}_{1}^{D}-V}_{2}^{D}$	${{V}_{1}^{D}-V}_{2}^{D}$	${{V}_{1}^{D}-H}_{2}^{D}$

	Photon 1-up; Photon 2-down				Photon 1-dowon; Photon 2-up			
${E}_{A}$	${H}_{2}^{D}$	$0$	${{V}_{1}^{U}-H}_{2}^{D}$	${V}_{1}^{U}$	${H}_{1}^{D}$	${{H}_{1}^{D}-V}_{2}^{U}$	$0$	${V}_{2}^{U}$
${E}_{B}$	${H}_{1}^{U}$	${{H}_{1}^{U}-V}_{2}^{D}$	$0$	${V}_{2}^{D}$	${H}_{2}^{U}$	$0$	${{V}_{1}^{D}-H}_{2}^{U}$	${V}_{1}^{D}$

	Photon 1-up; Photon 2-up				Photon 1-down; Photon 2-down			
${E}_{A}$	${V}_{2}^{U}$	$0$	${{V}_{1}^{U}-V}_{2}^{U}$	${V}_{1}^{U}$	${H}_{1}^{D}$	${{H}_{1}^{D}-H}_{2}^{D}$	$0$	${H}_{2}^{D}$
${E}_{B}$	${H}_{1}^{U}$	${{H}_{1}^{U}-H}_{2}^{U}$	$0$	${H}_{2}^{U}$	${V}_{2}^{D}$	$0$	${{V}_{1}^{D}-V}_{2}^{D}$	${V}_{1}^{D}$

The narrow-linewidth laser L is intensity attenuated for a low mean photon number, whose Poisson-distributed single-photon rate satisfies individual and independent statistics in measurements. For spectral bandwidth $2\Delta$, an AOM is inserted in each arm of the first NMZI in a double-pass scheme, as shown in the Inset of Fig. 1a, where both AOMs are synchronized and oppositely scanned. For a given spectral bandwidth of AOMs, the diffracted photons roughly satisfy a Gaussian-like profile $\Delta$, as shown in Fig. 1b. To satisfy random detuning at $\pm\updelta {f}_{j}$ for a *j*th photon pair, the AOM’s scan rate is set to be faster than the resolving time of the single photon detector or the inverse of the mean photon number, satisfying random measurements. As a result, the output photon pairs of the NMZI result in 16 different polarization-basis combinations, whose photon characteristics are distinguishable, resulting in no interference fringe. By a followed PBS in each output port of the NMZI, transparent and reflected photons are separated into horizontal and vertical polarization groups, respectively. This linear optics-based polarization-basis separation of coherent photon pairs is critical to the present coherence method to accomplish the quantum feature, mimicking the degenerate type I entangled photon pairs from SPDC^21,22^.

Table 1 shows all possible polarization-basis combinations of the paired photons in Fig. 1. By definition of the coincidence detection, only doubly-bunched photons are considered with a ~ 1% error rate resulting from higher-order bunched photons^35^. By the first BS of the NMZI, four possible photon-path choices are randomly allocated to each photon pair. In each photon-path choice, four different polarization-basis combinations are given randomly, resulting in a total of 16 path-polarization combinations for each pair of photons 1 and 2 (see two charts from the top). By the action of consecutive PBSs in both output paths of the first NMZI, single-path propagating photon pairs are automatically excluded from measurements (see the second and last chart). By the last PBS, both-path propagating photon pairs are separated into either orthogonally polarized or the same-polarized photon groups (see the third chart). Eventually, polarization-basis controlled photon pairs are individually tested for quantum features of the HOM effects by the last BS^14^. In Fig. 1a, the superscript of the polarization basis indicates a corresponding up (U) or down (D) path of the first NMZI. The subscript indicates the photon number in each pair, which cannot be discernable by Poisson distribution.

Table 2 shows the final sets of PBS-caused polarization-basis control for coincidence measurements in Fig. 1. By the polarization-basis separation analyzed in Table 1, the same polarization-basis sets, e.g. H–H (V–V) is independently grouped for coincidence measurements, as shown in the red- (blue-) shaded regions for detectors D1 and D3 (D2 and D4). These same-polarization-basis sets of photons satisfy the opposite frequency relation in each pair, as shown in Fig. 1b, corresponding to the signal and idler photons from SPDC. The number ‘1’ in the shaded regions indicates the perfect correlation between paired photons regardless of the frequency detuning in each set (see “Analysis” section). Due to coherence, however, the cross-correlation between the orthogonal polarization-basis sets of photons also exists, as denoted by superscript $\updelta$ in the off-diagonal direction. In this case, the same frequency photons are grouped in each pair. Between shaded and unshaded groups, simultaneous measurements are not allowed due to coincidence detection. The same detuned pair between D1 and D3 is also possible if two photons propagate along the same path until the last BS (see the green pairs in Table 1). This event is however eliminated by the heterodyne detection of the coincidence measurements. Thus, the present method of coherently driven quantum features using a linear optics-based polarization-basis control applies only for both shaded and unshaded regions separately. In the Analysis, the same polarization-basis groups of paired photons are considered.

**Table 2:**
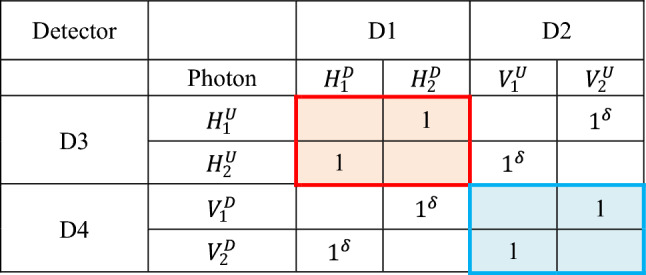
An entangled pair chart for Fig. 1.

The subscript ‘D’ and ‘U’ indicates $-\delta f$ and $\delta f$, respectively, as shown in Fig. 1b. ‘1’ indicates entanglement between symmetrically (oppositely) photon detuned pairs in Fig. 1b, whereas ‘${1}^{\delta }$’ is for the same frequency photons.

## Analysis

For Fig. 1, we derive coherence solutions of two-photon quantum features via coincidence detection between two output photons measured by single photon detectors D1 and D3. By definition of doubly-bunched photons and coincidence detection, simultaneous measurements between different color sets in Table 2 are not possible. At a low mean photon number, the ratio of doubly-bunched photons to single photons is ~ 1%^35^. Similarly, the ratio of higher-order bunched photons to the doubly-bunched photons is ~ 1%^35^. The coincidence detection eliminates both single photon and vacuum states from measurements^35^. Thus, the statistical error of coincidence measurements in Fig. 1 is ~ 1%, which is negligible. This kind of statistical error is inevitable for any type of spontaneous emission process including SPDC.

From Table 2, the photon numbers 1 and 2 cannot be discernable due to identical particles given by Boson characteristics of Poisson distribution. Thus, the NMZI output photons can be represented for the *j*th pair as:

1 $${\mathrm{E}}_{A}^{j}=\frac{{E}_{0}}{\sqrt{2}}\left(-{V}^{U}{e}^{i(\varphi \pm\updelta {f}_{j}t)}+{H}^{D}{e}^{\mp i\updelta {f}_{j}t}\right),$$

2 $${\mathrm{E}}_{B}^{j}=\frac{i{E}_{0}}{\sqrt{2}}\left({H}^{U}{e}^{i(\varphi \pm\updelta {f}_{j}t)}+{V}^{D}{e}^{\mp i\updelta {f}_{j}t}\right),$$

where ${H}^{U}$ $({H}^{D})$ stands for the horizontal polarization basis of a UP (DOWN)-path propagating photon. Likewise, ${V}^{U}$ $({V}^{D})$ stands for the vertical polarization basis of a UP (DOWN)-path propagating photon in the NMZI. In addition to the synchronized opposite-frequency scanning by a set of AOMs, a phase $\mathrm{\varphi }$ controller, e.g., a piezo-electric transducer (PZT) is added to the UP-path propagating photons for the first NMZI. Here, the PZT-induced phase should be dependent upon $\updelta {f}_{j}$, resulting in ${\mathrm{\varphi }}_{j}$. For simplicity, thus, the PZT-induced phase is replaced by $\mathrm{\varphi }\pm\updelta {f}_{j}\mathrm{t}\to \pm\updelta {f}_{j}{\tau }_{1}(\varphi )$, where ${\tau }_{1}$ is the $\mathrm{\varphi }$-induced time delay in the first NMZI. Due to no interaction between orthogonal polarization bases in Eqs. (1) and (2)^38,39^, the corresponding mean intensities become $\bigg\langle {I}_{A}\bigg\rangle =\bigg\langle {I}_{B}\bigg\rangle =\bigg\langle {I}_{0}\bigg\rangle$, where ${I}_{0}={E}_{0}{E}_{0}^{*}$, and ${E}_{0}$ is the single photon amplitude.

In the second NMZI, the phase $\uppsi$ is applied to ${E}_{A1}$ and ${E}_{B4}$, where these photons are from the DOWN path of the first NMZI. Like $\updelta {f}_{j}{\tau }_{1}(\varphi )$, the $\uppsi$-induced phase is represented by $\updelta {f}_{j}{\tau }_{2}(\psi )$, where ${\tau }_{2}$ is the $\uppsi$-induced time delay in the second NMZI. Thus, photon amplitudes used for the coincidence detection are finally represented by ${\mathrm{E}}_{A1}^{j}=\frac{{E}_{0}}{\sqrt{2}}{H}^{D}{e}^{\mp i\updelta {f}_{j}{\tau }_{2}}$, ${\mathrm{E}}_{A2}^{j}=\frac{-i{E}_{0}}{\sqrt{2}}{V}^{U}{e}^{\pm i\updelta {f}_{j}{\tau }_{1}}$, ${\mathrm{E}}_{B3}^{j}=\frac{i{E}_{0}}{\sqrt{2}}{H}^{U}{e}^{\pm i\updelta {f}_{j}{\tau }_{1}}$, and ${\mathrm{E}}_{B4}^{j}=\frac{-{E}_{0}}{\sqrt{2}}{V}^{D}{e}^{\mp i\updelta {f}_{j}{\tau }_{2}}$.

To verify the quantum feature of the two-photon correlation in Fig. 1, a conventional method of the Hong-Ou-Mandel effect is adapted for the interacting photon pairs on the BS. The amplitudes of the output photons from the BS are as follows:

3 $${\mathrm{E}}_{1}^{j}=\frac{1}{\sqrt{2}}\left(i{\mathrm{E}}_{A1}^{j}+{\mathrm{E}}_{B3}^{j}\right)=\frac{i{E}_{0}}{2}\left({H}^{D}{e}^{\mp i\updelta {f}_{j}{\tau }_{2}}+{H}^{U}{e}^{\pm i\updelta {f}_{j}{\tau }_{1}}\right),$$

4 $${\mathrm{E}}_{2}^{j}=\frac{1}{\sqrt{2}}\left(i{\mathrm{E}}_{A2}^{j}+{e}^{i\psi }{\mathrm{E}}_{B4}^{j}\right)=\frac{{E}_{0}}{2}\left({V}^{U}{e}^{\pm i\updelta {f}_{j}{\tau }_{1}}-{V}^{D}{e}^{\mp i\updelta {f}_{j}{\tau }_{2}}\right),$$

5 $${\mathrm{E}}_{3}^{j}=\frac{1}{\sqrt{2}}\left({\mathrm{E}}_{A1}^{j}{e}^{i\psi }+i{\mathrm{E}}_{B3}^{j}\right)=\frac{{E}_{0}}{2}\left({H}^{D}{e}^{\mp i\updelta {f}_{j}{\tau }_{2}}-{H}^{U}{e}^{\pm i\updelta {f}_{j}{\tau }_{1}}\right),$$

6 $${\mathrm{E}}_{4}^{j}=\frac{1}{\sqrt{2}}\left({\mathrm{E}}_{A2}^{j}+i{e}^{i\psi }{\mathrm{E}}_{B4}^{j}\right)=\frac{-i{E}_{0}}{2}\left({V}^{U}{e}^{\pm i\updelta {f}_{j}{\tau }_{1}}+{V}^{D}{e}^{\mp i\updelta {f}_{j}{\tau }_{2}}\right).$$

Thus, the corresponding mean intensities are calculated as:

7 $$\langle {I}_{1}\rangle =\frac{\langle {I}_{0}\rangle }{4} \bigg\langle \sum_{j}\left({H}^{D}{e}^{\mp i\updelta {f}_{j}{\tau }_{2}}+{H}^{U}{e}^{\pm i\updelta {f}_{j}{\tau }_{1}}\right)\left({H}^{D}{e}^{\pm i\updelta {f}_{j}{\tau }_{2}}+{H}^{U}{e}^{\mp i\updelta {f}_{j}{\tau }_{1}}\right)\bigg\rangle =\bigg\langle \frac{{I}_{0}}{2}\bigg\rangle \bigg\langle \sum_{j}\left[1+\mathrm{cos}(2\updelta {f}_{j}({\tau }_{1}+{\tau }_{2}))\right]\bigg\rangle ,$$

8 $$\langle {I}_{2}\rangle =\frac{\langle {I}_{0}\rangle }{4}\bigg\langle \sum_{j}\left({V}^{U}{e}^{\pm i\updelta {f}_{j}{\tau }_{1}}-{V}^{D}{e}^{\mp i\updelta {f}_{j}{\tau }_{2}}\right)\left({V}^{U}{e}^{\mp i\updelta {f}_{j}{\tau }_{1}}-{V}^{D}{e}^{\pm i\updelta {f}_{j}{\tau }_{2}}\right)\bigg\rangle = \bigg\langle \frac{{I}_{0}}{2}\bigg\rangle \bigg\langle \sum_{j}\left[1-\mathrm{cos}(2\updelta {f}_{j}({\tau }_{1}+{\tau }_{2}))\right]\bigg\rangle ,$$

9 $$\langle {I}_{3}\rangle =\frac{\langle {I}_{0}\rangle }{4}\bigg\langle \sum_{j}\left({H}^{D}{e}^{\mp i\updelta {f}_{j}{\tau }_{2}}-{H}^{U}{e}^{\pm i\updelta {f}_{j}{\tau }_{1}}\right)\left({H}^{D}{e}^{\pm i\updelta {f}_{j}{\tau }_{2}}-{H}^{U}{e}^{\mp i\updelta {f}_{j}{\tau }_{1}}\right)\bigg\rangle =\bigg\langle \frac{{I}_{0}}{2}\bigg\rangle \bigg\langle \sum_{j}\left[1-\mathrm{cos}(2\updelta {f}_{j}({\tau }_{1}+{\tau }_{2}))\right]\bigg\rangle .$$

10 $$\langle {I}_{4}\rangle =\frac{\langle {I}_{0}\rangle }{4}\bigg\langle \sum_{j}\left({V}^{U}{e}^{\pm i\updelta {f}_{j}{\tau }_{1}}+{V}^{D}{e}^{\mp i\updelta {f}_{j}{\tau }_{2}}\right)\left({V}^{U}{e}^{\mp i\updelta {f}_{j}{\tau }_{1}}+{V}^{D}{e}^{\pm i\updelta {f}_{j}{\tau }_{2}}\right)\bigg\rangle =\bigg\langle \frac{{I}_{0}}{2}\bigg\rangle \bigg\langle \sum_{j}\left[1+\mathrm{cos}(2\updelta {f}_{j}({\tau }_{1}+{\tau }_{2}))\right]\bigg\rangle .$$

Unlike a conventional laser interference case, Eqs. (7)–(10) show a propagation-distance proportional phase shift due simply to the opposite detuning $\pm\updelta {f}_{j}{\tau }_{k}$, where ${\tau }_{k}$ is a path-length dependent transit time. Here, it should be noted that the coincidence time between the paired photons is for ${\tau }_{1}={\tau }_{2}$, where $2\updelta {f}_{j}\left({\tau }_{1}+{\tau }_{2}\right)\gg 1$. Thus, $\langle 1+\mathrm{cos}(2\updelta {f}_{j}({\tau }_{1}+{\tau }_{2}))\rangle =1$, satisfying the uniform local intensities $\langle {I}_{k}\rangle =\frac{\langle {I}_{0}\rangle }{2}$.

The coincidence detection between two output photons ${E}_{1}$ and ${E}_{3}$ is not like the local intensity product between Eqs. (7) and (9) because of the incompatible basis products for the same path of NMZI, as shown in Table 2:

11 $$\langle {R}_{13}\left(0\right)\rangle = \bigg\langle \sum_{j}{\mathrm{E}}_{1}^{j}{\mathrm{E}}_{3}^{j}(cc)\bigg\rangle =\frac{ \langle {I}_{0}^{2}\rangle }{16}\bigg\langle \sum_{j}\left({H}^{D}{e}^{\mp i\updelta {f}_{j}{\tau }_{2}}+{H}^{U}{e}^{\pm i\updelta {f}_{j}{\tau }_{1}}\right)\left({H}^{D}{e}^{\mp i\updelta {f}_{j}{\tau }_{2}}-{H}^{U}{e}^{\pm i\updelta {f}_{j}{\tau }_{1}}\right)(cc)\bigg\rangle =\frac{\langle {I}_{0}^{2} \rangle }{16}{H}^{D}{H}^{U}\bigg\langle \sum_{j}\left(-{e}^{\mp i\updelta {f}_{j}{\tau }_{21}}+{e}^{\mp i\updelta {f}_{j}{\tau }_{21}}\right)(cc)\bigg\rangle =0,$$

where cc is a complex conjugate, ${\tau }_{21}={\tau }_{2}-{\tau }_{1}$, and ${H}^{k}{H}^{k}=0$. Likewise, the coincidence detection between photons ${E}_{2}$ and ${E}_{4}$ is as follows:

12 $$\langle {R}_{24}\left({\tau }_{21}\right)\rangle =\bigg\langle \sum_{j}{\mathrm{E}}_{2}^{j}{\mathrm{E}}_{4}^{j}(cc)\bigg\rangle =\frac{\langle {I}_{0}^{2} \rangle }{16}\bigg\langle \sum_{j}\left({V}^{U}{e}^{\pm i\updelta {f}_{j}{\tau }_{1}}-{V}^{D}{e}^{\mp i\updelta {f}_{j}{\tau }_{2}}\right)\left({V}^{U}{e}^{\pm i\updelta {f}_{j}{\tau }_{1}}+{V}^{D}{e}^{\mp i\updelta {f}_{j}{\tau }_{2}}\right)(cc)\bigg\rangle =\frac{\langle {I}_{0}^{2}\rangle }{16}{V}^{D}{V}^{U}\bigg\langle \sum_{j}\left(-{e}^{\mp i\updelta {f}_{j}{\tau }_{21}}+{e}^{\mp i\updelta {f}_{j}{\tau }_{21}}\right)(cc)\bigg\rangle =0.$$

Unlike uniform local intensities in Eqs. (7)–(10), the two-photon correlation in Eqs. (11) and (12) for the coherently manipulated polarization basis show the quantum feature of anti-correlation. In the coincidence counting module, the coincidence detection cross-correlation between the single-photon detector-generated electrical pulses whose pulse duration is a few ns. Due to the Gaussian-like spectral distribution in Fig. 1b, the single photon-induced electrical pulse should show a similar probability distribution, resulting in a Gaussian-like cross-correlation as a function of ${\tau }_{21}$^40^. The sideband oscillation of the HOM dip is from this kind of cross-correlation.

## Discussion

In Eqs. (11) and (12), the time delay ${\tau }_{21}$ induced by $\psi$ and $\varphi$ is in the order of ${\Delta }^{-1}$. Unlike local intensities in Eqs. (7)–(10), each time delay of ${\tau }_{1}$ or ${\tau }_{2}$ is in the order of the laser’s coherence time which is much longer than ${\Delta }^{-1}$. Compared with a recent coherence study of the HOM effects for entangled photons^17^, Eqs. (11) and (12) show that the origin of the anticorrelation is in the definite phase shift $\frac{\pi }{2}$ between the paired photons regardless of their spectral detuning. The random phase between photon pairs given by either Poisson statistics or the SPDC process does not deteriorate the HOM effects due to independent measurements. The same fixed sum-phase relation of the paired photons is accomplished by the first BS of the NMZI in Fig. 1. Unlike local intensities in Eqs. (7)–(10), no ensemble decoherence effect is shown in Eqs. (11) and (12) due to the selective polarization-basis products.

The linear optics-based basis selection process is the key to the quantum feature derived in Eqs. (11) and (12), resulting in the second-order quantum superposition between selected basis products of interacting photons^18^. Without coincidence detection, such a measurement-event selection process cannot be possible due to the long coherence of each photon, allowing the cross-correlation between shaded and unshaded regions in Table 2. Thus, the resolving time of a photodetector plays an important role in coincidence detection, where this time scale must be shorter than the single photon rate. As a result, the quantum feature derived in Eqs. (10) and (11) must be limited to a microscopic regime of single photons as usually understood in quantum information science. For this, keeping a low mean-photon number is a technical requirement.

The advantage of the proposed method is in the practical applications based on robust MZI with an active phase stabilization technique^34^. To solve the major error on MZI caused by air turbulence, the MZI phase stabilization technique has been a common technique in conventional sensing areas such as ring laser gyroscope as well as gravitational wave detection. A potential application of the proposed idea is for a macroscopic entanglement, where the coherence manipulation of polarization-frequency correlation using AOMs is the major huddle for the selective measurement process (discussed elsewhere).

## Conclusion

Coherently driven quantum features of the HOM effects were analyzed for the fundamental physics of quantum mechanics using linear optics-based polarization basis control of coherent photons. Unlike common understanding, the impossible quantum entanglement creation using coherent photons was analyzed for coherence manipulations of polarization-basis separation using heterodyne signals. Due to the intrinsic coherence property of mixed states, the action of the polarization-basis control by a set of PBSs resulted in an inevitable 50% loss of measurement events. As a result, coherently induced HOM-type anticorrelation, i.e., the photon bunching phenomenon on a BS, was derived from polarization-basis modified coherent photon pairs via coincidence detection, regardless of the bandwidth. Due to the linear optics-based coherence approach, the proposed method of coherently driven HOM effects should set a new course in quantum mechanics. This work may give a step toward macroscopic entanglement generation in the future, even though such a phenomenon seems to be impossible due to mutual coherence among interacting photons at the present scope.

## Data Availability

All data generated or analysed during this study are included in this published article.
